# The extravascular compartment of the bone marrow: a niche for *Plasmodium falciparum* gametocyte maturation?

**DOI:** 10.1186/1475-2875-11-285

**Published:** 2012-08-20

**Authors:** Eric Farfour, Frédéric Charlotte, Catherine Settegrana, Makoto Miyara, Pierre Buffet

**Affiliations:** 1 Laboratoire de parasitologie-mycologie, Assistance publique hôpitaux de Paris, Groupe Hospitalier Pitié-Salpêtrière, 47 Boulevard de l'Hôpital, 75651, Paris Cedex 13, France; 2 Laboratoire d'anatomie-pathologie, Assistance publique hôpitaux de Paris, Groupe Hospitalier Pitié-Salpêtrière, Paris, France; 3 Service de médecine interne 2, Assistance publique hôpitaux de Paris, Groupe Hospitalier Pitié-Salpêtrière, Paris, France; 4 Département d’immunologie, Assistance publique hôpitaux de Paris, Groupe Hospitalier Pitié-Salpêtrière, Paris, France; 5 Laboratoire d’hématologie, Assistance publique hôpitaux de Paris, Groupe Hospitalier Pitié-Salpêtrière, Paris, France; 6 Unité Prévention et Thérapie Moléculaire des Maladies Humaines, CNR des corynébactéries du complexe diphtheriae, Institut Pasteur, 25-28 rue du Dr Roux, 75014, Paris, France; 7 Unité d'Immunologie Moléculaire des Parasites, Institut Pasteur, Paris, France; 8 Inserm-UPMC, Paris 6 Université, UMRs945, 91 boulevard de l’hôpital, 75013, Paris, France

**Keywords:** Malaria, Transmission, Gametocyte, Bone marrow, *Plasmodium falciparum*

## Abstract

**Background:**

*Plasmodium falciparum* immature gametocytes accumulate in the bone marrow, but their exact location in this tissue remains unclear.

**Methods:**

The stage and deposition pattern of gametocytes was analysed on histological sections of a bone marrow sample collected in a patient with subacute *P. falciparum* malaria.

**Results:**

A majority (89%) of immature stages II to IV gametocytes and a minority (29%) of mature stage V gametocytes were observed in extravascular spaces.

**Discussion and conclusion:**

These observations represent a valuable step towards understanding sequestration patterns of *P. falciparum* gametocytes and may ultimately lead to novel transmission-blocking interventions.

## Background

*P. falciparum* gametocytes (human erythrocytes infected with sexual-stage of *P. falciparum*) are essential for malaria transmission. However, knowledge on the fine interactions between gametocytes and human tissues is limited [1]. Because erythrocytes circulate in vessels, *P. falciparum*-infected erythrocytes are predicted to remain in the intravascular compartment. Erythrocytes infected with mature asexual stages (mature trophozoites and schizonts) are sequestered in the lumen of small vessels via their adhesion to microvascular endothelial cells [2]. Immature gametocytes are similarly absent from large vessels, but are present in the bone marrow. Smalley *et al.* have shown that in *P. falciparum* infected children, the density of immature gametocytes is 10-fold greater on bone marrow smears than on peripheral blood smears [3], however their exact location in the bone marrow has not been described.

### Case presentation

A 42-year-old male patient was admitted to a peripheral hospital for headache and fever (39°C) 15 days after returning from Cameroon, where he had spent two months under suboptimal anti-malarial prophylaxis with doxycycline. Microbiological explorations, including thin and thick blood smears, were initially negative. However, the fever persisted and the haemoglobin level fell to 92g/L. The patient was referred to a university hospital with suspicion of a macrophage activation syndrome. A venous blood smear was performed on admission (i e, 10 days after onset of symptoms) and came back positive for *P. falciparum* (0.6% parasitaemia). A three-day course of oral atovaquone plus proguanil was effective, as assessed by fever clearance and disappearance of asexual parasite stages from subsequent blood smears. Few mature stage V gametocytes were observed on thick blood smears at day 3 and day 7. A bone marrow biopsy was performed after two days of treatment to exclude other causes of fever and anaemia.

**Figure 1:**
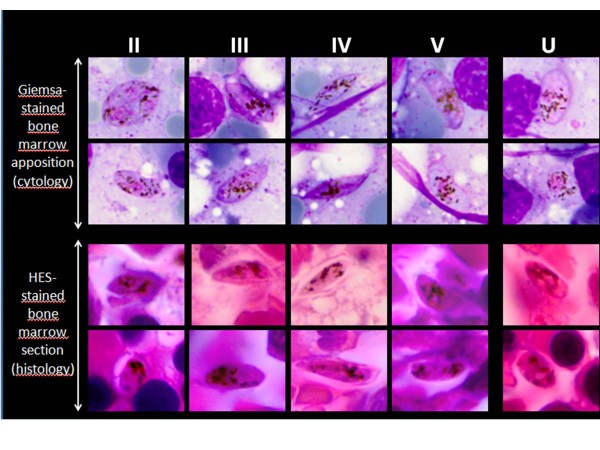
**Gametocyte staging on Giemsa-stained smears (cytology, reference method) and on HES-stained sections (histology) from the same bone marrow biopsy sample described in this report.** Leaf-shaped forms, hemi-ellipsoid forms, forms with at least one sharp extremity, and crescent forms with two rounded extremities, a concave and a convex border were counted as stage **II**, **III**, **IV** and **V**, respectively (columns **II-V**). A form with finely distributed pigment, but not falling into any of the previous categories, was categorized as undetermined stage (column **U**). Counting results appear on Table 1.

**Figure 2:**
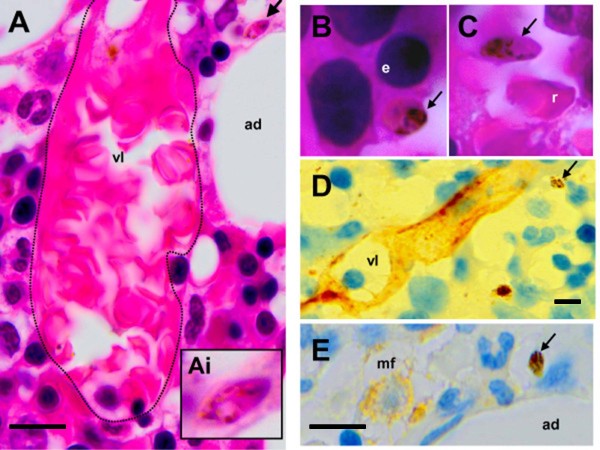
**A (arrow and Ai insert) and B: HES-stained bone marrow sections showing immature gametocytes - elongated shape, pink and purple staining, finely distributed pigment – outside small vessels (vl) and in close contact with resident cells such as adipocytes (ad) or erythroblasts (e).** The dotted line in A highlights the vessel wall. **C:** Mature stage V gametocyte close to a mature red blood cell (C, r) in the lumen of a vessel. **D** and **E:** Immunohistochemistry (antibodies revealed with peroxydase) showing gametocytes (arrows) distant from CD34-positive vascular structures (vl) or CD68-positive macrophages (mf).

Stained histological sections of the bone marrow showed gametocytes at different maturation stages and numerous macrophages containing blood cells or malaria pigment. Gametocytes were readily identified and staged based on their oval or elongated shape and finely distributed pigment (Figure 1). Only 29% (4/14) of stage V gametocytes were in extravascular spaces (Table 1), the majority being in intravascular spaces (Figure 2C). By contrast, 89% (87/98) of immature stage II-IV gametocytes were observed in extravascular spaces (Table 1, Figure 2) in immediate contact with erythroblasts (Figure 2B), adipocytes (Figure 2A) or other nucleated cells (Figure 2B). Several gametocytes did not co-localize with CD34-positive vascular structures (Figure 2D) or CD68-positive cells (Figure 2E).

**Table 1:** Number (%) of gametocytes of each stage in the different compartments of the bone marrow

	**Stage II**	**Stage III**	**Stage IV**	**Stage V**	**Undetermined**	**Total**
Intravascular	1	0	1	8	2	12 (10)
Extravascular	37	30	20	4	17	108 (67)
Unknown	5	3	1	2	8	19 (23)
Total	43 (31)	33 (24)	22 (16)	14 (10)	27 (19)	139
	88 (71)			14 (10)	27 (19)	

## Conclusion

The stage and deposition pattern of gametocytes as analysed on histological sections, including the absence of co-localization with CD34-positive vascular structures suggest that immature *P. falciparum* gametocytes undergo part of their development in the extravascular spaces of the bone marrow. This development may also occur in other organs, the exploration of which is not possible in patients. The absence of co-localization with CD68-positive cells also suggests that the extravascular location of gametocytes was not induced by migration of gametocyte-containing monocytes from the blood to the extra-vascular spaces. Like mature asexual *P. falciparum* blood stages (pigmented trophozoites and schizonts), immature gametocytes are absent from large vessels [4]. However, while mature asexual stages sequester in microvessels by adhering strongly to endothelial cells, immature gametocytes do not express high levels of known adhesins and are poorly adherent to endothelial cells *in vitro*[5,6]. Their presence in the extravascular compartment provides a new, convincing explanation for this apparent contradiction. Absence of immature gametocytes from the peripheral circulation may indeed result from their location in extravascular spaces rather than from adherence to endothelial cells in the lumen of microvessels. While asexual parasite maturation from early ring stages to mature trophozoites and schizonts takes 1 to 2 days, gametocytes maturation from stage I to stage V is much longer and takes approximately 10 days [1,7]. Therefore, extravascular spaces may provide a better niche for prolonged survival than the lumen of small vessels. Both mature asexual stages and immature gametocytes display a markedly increased stiffness as compared to that of uninfected red blood cells [8,9]*.* Sequestration of *P. falciparum-*infected red blood cells away from the general circulation is considered a survival strategy to escape mechanical filtration by the spleen [4,10,11]. Unlike immature gametocytes, mature gametocytes are deformable [8] and can, therefore, circulate without being retained in the spleen [10]. Drugs modifying the deformability of immature or mature gametocytes may influence their ability to squeeze and cross barriers between the intravascular and the extravascular compartments, as well as to flow through narrow splenic slits [8]. Mature gametocytes must circulate in the periphery to be taken up by Anopheles then be transmitted to human beings. Interventions reducing their deformability may induce their mechanical retention in the spleen, thus removing them from the peripheral circulation. Such interventions would have a transmission-blocking effect. In conclusion, this observation highlights a singular aspect of *P. falciparum* gametocyte sequestration, which may lead to a better understanding of gametocyte survival mechanisms and may ultimately contribute in the progress toward malaria eradication.

## Competing interests

The authors declare that they have no competing interests.

## Authors’ contributions

MM was the reference clinician attending the patient, performed the bone marrow biopsy, and reviewed the manuscript. CS performed the cytological analyses and reviewed the manuscript. FC performed pathological analyses, including immunochemistry of the bone marrow biopsy and reviewed the manuscript. EF and PB performed parasitological and pathological analyses and wrote the manuscript. All authors read and approved the final manuscript.
